# How does the self-reported health of undergraduate nursing students change during their degree programme? Survey results from a Scottish University

**DOI:** 10.1186/s12912-021-00563-w

**Published:** 2021-03-17

**Authors:** Josie M. M. Evans, Federico Andreis, Dawn M. Cameron, Claire E. Eades

**Affiliations:** Faculty of Health Sciences and Sport, University of Stirling, Stirling, Scotland FK9 4LA UK

## Abstract

**Background:**

The lifestyle behaviours, and the physical and mental health of nurses, are poorer than those of other allied health professionals, and of the general population. However, these were no less favourable among first year undergraduate nursing students at a Scottish Higher Education Institution (HEI) than among similar people of the same age. We compared health and health behaviours among the same cohort of undergraduate nursing students over the course of their degree.

**Methods:**

An anonymous self-complete repeat cross-sectional survey was administered during a timetabled teaching session at three time-points to undergraduate nursing students at the start of Years 1, 2 and 3 of their programme. They had received written information about the study previously.

**Results:**

Self-reported health did not change significantly over time, but there was a clear decline over the 3 years in the proportions of students rating their mental health as excellent/very good/good and a concomitant increase in those rating their mental health as fair/poor. Correspondingly, the mean WEMWBS wellbeing score declined over the 3 years, with the proportion of students with a score of < 46 (indicating either high risk of major depression, or in high risk of psychological distress and increased risk of depression) increasing from one quarter to one half. This effect was captured and described using a Bayesian regression analysis. The most noticeable change in health behaviours was a decline in physical activity levels over the study period. The proportion of students managing 150 min of weekly physical activity decreased from three quarters to two thirds. This was reflected in higher self-reported sedentary time, although there were no observable trends over time in mean BMI, or proportions of students categorised as overweight or obese.

**Conclusions:**

This paper suggests that there may be a decline in mental health and in participation in physical activity among nursing students over the course of their degree. We recommend the incorporation of an intervention into the undergraduate nursing curriculum that promotes and encourages regular physical activity, offering students the opportunity to learn about health promotion and lifestyle change in practice, to improve their own physical health, and to address mental wellbeing.

**Supplementary Information:**

The online version contains supplementary material available at 10.1186/s12912-021-00563-w.

## Background

Recent evidence suggests that the lifestyle behaviours, and the physical and mental health of nurses, are poorer than those of other allied health professionals, and even those of the general population [1]. However, we have shown that the health and health behaviours of first-year undergraduate nursing students at a Scottish Higher Education Institution (HEI) were no worse than those of similarly-aged respondents to the 2016 Scottish Health Survey [2]. But with the expanding health promotion role of nurses, it is important that they are credible role models to their patients, and a reasonable aspiration is that their health behaviour profile would be better than that of the general population. Their academic education surrounding health promotion, alongside exposure to the damaging health-related effects of poor lifestyle choices during clinical training, might result in improvement in the health behaviours of nursing students over time, but the academic and social demands of University life could conversely lead to a deterioration [3]. We have therefore continued to monitor the health and health behaviours of this cohort of undergraduate nursing students. The objective of this study was to compare these over time at three points during their programme.

## Methods

Undergraduate nursing students (mental health and adult branch) at our Scottish HEI were given Participant Information Leaflets about our study, then a paper-based self-report questionnaire was administered to them by an academic member of staff during timetabled face-to-face teaching sessions at least 48 h later. This occurred on three occasions at the start of each academic year (September 2016, 2017 and 2018). The questionnaire contained seven sections relating to physical and mental health, and important health behaviours. It was adapted from sections of questionnaires used in several other HEIs [4], with additional questions informed by the Scottish Health Survey [5], and had a combination of open and semi-structured questions. Widely used measures of self-rated general health were also incorporated into the questionnaire [6], in that students were asked to rate separately their general physical and mental health as either excellent, very good, good, fair or poor. Emotional wellbeing was assessed using the Warwick-Edinburgh mental wellbeing scale (WEMWBS). This provides a single score of between 14 and 70, derived from a 14 item scale. It was developed and validated in student populations [7] and had test-retest reliability of 0.83 [8]. We asked students to report their weight and height in any units, then we converted units where necessary and calculated body mass index (BMI) as kg/m^2^. A copy of the questions administered are presented in Appendix 1 (a supplemental file).

The students were asked not to give their name or any other information that would make them identifiable, although they generated their own, unique 12-digit alpha-numeric identifier to add to the questionnaire for linkage purposes [2]. In this way, the results were anonymous. Students who chose not to complete the questionnaire were asked to return a blank form. Data were analysed descriptively using the SPSS v21.0 programme (Chicago, Illinois) and R 3.6.1 [9]. Subjects with missing data were excluded from the relevant analyses, and numerical variables were checked for outliers. Proportions were determined for categorical variables, and mean, median, standard deviation and range derived for continuous variables.

We used the 12-digit identifier to link together results from the same students at different time-points and identified those for whom data were available for all 3 years. We constructed three mixed effect regression models for three measures that warranted further investigation to determine whether there was an effect over time: whether the student was a current smoker or not (logistic regression), whether the student self-reported at least 150 min of physical activity per week (logistic regression), and the WEMWBS score (simple linear regression). Independent variables included age, gender, whether the student was the first in their family to go to university and year of degree programme (first, second or third). All regression models were fitted using the R package rstanarm [10], and the usual convergence criteria were satisfied in all cases.

## Results

There were 207 questionnaires returned by first year students in 2016, 175 by second years in 2017 and 103 by third years in 2018. Students were predominately female, with proportions of 86, 73 and 84% each year respectively. Mean (median) ages were 24 (22) years, 25 (23) years and 27 (25) each year. From the numbers of student identified within each year group, the response rates were 88, 81 and 52% respectively. Table 1 presents summary results (results for Year 1 have been published previously [2]).

**Table 1:** Summary results for health and health behaviour measures for a cohort of undergraduate nursing students in the first, second and third years of their degree programme

	Year 1 (207 responses) n (%)	Year 2 (175 responses) n (%)	Year 3 (103 responses) n (%)
**General physical health**			
Excellent	12 (5.8)	4 (2.3)	7 (6.8)
Very good	36 (17.4)	28 (16.0)	19 (18.4)
Good	100 (48.3)	86 (49.1)	40 (38.8)
Fair	50 (24.2)	49 (28.0)	30 (29.1)
Poor	8 (3.9)	7 (4.0)	6 (5.8)
**Total**	206	174	102
**Mental Health**			
Excellent	19 (9.2)	12 (6.9)	6 (5.8)
Very good	81 (39.1)	50 (28.6)	21 (20.4)
Good	83 (40.1)	70 (40.0)	33 (32.0)
Fair	19 (9.2)	29 (16.6)	34 (33.0)
Poor	5 (2.4)	11 (6.3)	6 (5.8)
**Total**	207	172	99
	*n* = 198	*n* = 173	*n* = 96
WEMWBS score < 46	48 (24.2)	60 (34.7)	49 (51.0)
Mean (median) WEMWBS score	50.9 (50.9)	49.0 (50.0)	44.8 (45.0)
**Physical activity**			
**≥** 150 mins/week	157 (76.2)	143 (81.7)	65 (63.1)
Mean (median) sedentary hours			
Weekday	6.0 (6.0)	6.1 (6.0)	8.9 (8.0)
Weekend	6.4 (6.0)	6.2 (5.5)	8.0 (8.0)
**Diet**			
Very healthy	1 (2.9)	3 (1.7)	3 (2.9)
Healthy	26 (21.3)	43 (24.6)	33 (32.0)
Average	63 (58.5)	101 (57.7)	46 (44.7)
Unhealthy	19 (15.9)	26 (14.9)	21 (20.4)
Very unhealthy	0 (1.4)	2 (1.1)	–
Total	207	175	103
Breakfast most days	142 (68.9)	108 (61.7)	56 (54.4)
≥ 5 fruit/veg per day	25 (12.1)	22 (12.6)	15 (14.6)
Takeaway at least once/week	88 (42.7)	75 (42.9)	41 (39.8)
**BMI**	*n* = 165	*n* = 133	*n* = 84
Mean (median)	25.2 (24.1)	24.8 (24.1)	25.4 (24.2)
BMI 25–30	48 (29.0%)	37 (27.8%)	22 (26.1%)
(overweight) BMI ≥ 30 (obese)	30 (18.2)	19 (14.3)	16 (19.0)
**Sleep**			
Very well	35 (16.9)	33 (18.9)	19 (18.4)
Well	71 (34.3)	43 (24.6)	27 (26.2)
Average	79 (38.2)	70 (40.0)	39 (37.9)
Badly	21 (10.1)	23 (13.1)	14 (13.6)
Very badly	1 (0.5)	6 (3.4)	4 (3.9)
Total	207	175	103
< 7 h sleep/night	70 (34.0)	63 (36.0)	44 (42.7)
Mobile device > 30 mins	129 (62.3)	99 (56.6)	66 (64.1)
**Alcohol**	*n* = 204	n = 173	*n* = 100
Consumes any alcohol	186 (92.1)	154 (89.0)	86 (86.0)
Drinks alcohol			
occasionally/never	134 (65.7)	115 (66.5)	78 (78.0)
Drinks ≥14 units/wk	12 (5.9)	16 (9.2)	3 (3.0)
Weekly binge drinking	32 (15.7)	35 (20.2)	15 (15.0)
**Smoking**			
Daily	32 (15.8)	16 (9.1)	9 (8.7)
< daily	18 (8.9)	22 (12.6)	11 (10.7)

In relation to physical health, a higher proportion of students in Year 3 (25.2%) rated their health as excellent or very good compared to proportions within the two previous year groups (23.2% in Year 1 and 18.3% in Year 2). However, only 38.8% rated their health as good, compared to 48.3% in Year 1 and 49.1% in Year 2. A higher proportion in Year 3 (34.9%) rated their health as fair/poor, compared to 28.1% in Year 1 and 32.0% in Year 2.

This contrasted with results for mental health. There was a clear decline over the 3 years in the proportions of students rating their mental health as excellent/very good/good (from 48.3% in Year 1 to 26.2% in Year 3) and a concomitant increase in those rating their mental health as fair/poor (from 11.6% in Year 1 to 38.8% in Year 3). This result was backed up by the results of the WEMWBS score. Not only did the mean score decline from 50.9 to 44.8 over the 3 years, but the proportion of students with a WEMWBS score of < 46 (indicating either high risk of major depression, or in high risk of psychological distress and increased risk of depression) increased from 24.1% in Year 1 to 51.0% in Year 3.

Health behaviours were also investigated. Physical activity levels declined over the study period, with the proportion of students managing 150 min of physical activity per week decreasing from 76.2% in Year 1 to 63.1% in Year 3. This was reflected in higher self-reported sedentary time. The results for dietary behaviour were less clear cut. Fewer students over time reported their diets as average (58.5% in Year 1 compared with 44.7% in Year 3), with higher proportions over time rating their diets as either healthy or very healthy, or as unhealthy or very unhealthy. There was a decrease in the proportion of students eating breakfast most days, from 68.9% in Year 1 to 54.4% in Year 3 (but no difference for consumption of fruit and vegetables, and takeaways). However, there were no clear differences over time in mean BMI, or proportions of students with a BMI categorising them as overweight or obese.

There was a small increase over time in the proportions of students who reported sleeping poorly or very poorly (from 10.6% in Year 1 to 17.5% in Year 3), and there were higher proportions of students who reported sleeping for less than seven hours per night (from 34.0% in Year 1 to 42.7% in Year 3). There was evidence of a possible decline in alcohol consumption over time, with any alcohol consumption declining from 92.1% in Year 1 to 86.0% in Year 3, and an increasing proportion of students drinking alcohol only occasionally or never. Binge drinking appeared to peak in Year 2. The proportion of students smoking (current or occasional) decreased from 24.7 to 19.4% over the course. The decline was most marked for current smokers; whereas occasional smoking was higher in Year 2, perhaps indicating that some smokers were cutting down and moving from daily to occasional smoking.

We identified 51 students for whom data was available for all three time-points. The results of the regression analyses, using a Bayesian approach, are presented in Table 2. They suggest a clear effect of worsening WEMWBS score over time, but no effect for smoking or physical activity behaviour. The other notable result was that males had poorer WEMWBS scores. However, among this sub-sample of 51 students, the proportion of smokers (15%) was lower, and the proportion achieving 150 min of physical activity (84%) was higher than in the overall sample during the first year. In contrast, their mean WEMWBS score as baseline was very similar to that of the overall cohort.

**Table 2:** Mean, standard deviation, 10th, 50th, and 90th percentiles of the posterior distribution of the parameters estimates for the three regression models, constructed for 51 students for whom data were available at all three time-points

	Mean	Standard deviation	10%	50%	90%
**Dependent variable: Non-smoking**					
Age	0.1	0.2	−0.2	0.1	0.3
Not female *v* female	−1.6	2.3	−4.5	−1.6	1.3
Not first gen. Uni *v* first gen. Uni	1.7	1.8	−0.8	1.7	4.0
Year 2 *v* Year 1	0.0	0.3	−0.4	0.0	0.4
Year 3 *v* Year 1	0.0	0.3	−0.4	0.0	0.4
**Dependent variable: Self-reporting 150 min of PA per week**					
Age	0.1	0.2	− 0.3	0.1	0.1
Not female *v* female	−0.3	2.0	−2.9	−0.3	2.3
Not first gen Uni *v* first gen. Uni	0.1	1.6	−1.9	0.1	2.1
Year 2 *v* Year 1	0.0	0.2	−0.3	0.0	0.3
Year 3 *v* Year 1	0.0	0.2	−0.3	0,0	0.3
**Dependent variable: WEMWBS score**					
Age	0.2	0.2	−0.1	0.2	0.5
Not female *v* female	−6.4	4.6	−12.4	−6.4	−0.4
Not first gen. Uni *v* first gen. Uni	1.5	2.8	−2.2	1.4	5.0
Year 2 *v* Year 1	− 2.6	0.4	−3.1	− 2.6	− 2.1
Year 3 *v* Year 1	−7.0	0.3	−7.5	−7.0	−6.6

## Discussion

Among a cohort of undergraduate nurses who commenced their nursing training at a HEI in Scotland, a clear picture emerges of students experiencing deteriorating mental health over the course of their degree, and a decline in physical activity. Results for other health behaviours and associated outcomes were less clear cut, although alcohol consumption and smoking declined over the study period. Perhaps the most concerning result are the high proportions of students, increasing steadily over the course of the programme, self-reporting poor or very poor mental health. By the beginning of their third year, half of all students had a WEMWBS score indicative of a risk or high risk of major depression, or high risk of psychological distress. While, it could be that the survey was administered at a particularly stressful point in the programme, the clear trend over time belies this. The results of a linear regression analysis among a subgroup of 51 students confirm that the data are consistent with a real effect over time. There may therefore be a need for more targeted monitoring and support for the mental health of undergraduate nursing students. This is an international concern, and has prompted a study following up stress among nursing students in the US as they transition to their first job [11]. Potential poor psychological health among the nursing workforce is extremely worrying, given that they are likely to have longer careers than their predecessors, and require psychological capacity and resilience to provide high standards of nursing care.

The other health behaviour measure for which there was some evidence of significant worsening over time in the cross-sectional results was physical activity, but this was not evident among the subgroup of 51 students for whom data were available at all three time-points. Neither was it reflected in results for BMI, although there were unlikely to be substantial changes within the limited time frame of this study. Despite this, the current level of overweight/obesity among nurses in Scotland is 69% [12] and it is worrying that a decline in physical activity is already evident among students before they have even left further education. Other studies among nursing students in the US [13], Spain and Colombia [14], Hong Kong [15] and Thailand [16] have also found the majority of students not meeting physical activity guidelines. We have carried out qualitative research among second year student nurses who report that they find it increasingly difficult to build physical activity into their daily lives, given the time pressures of studying, working and attending clinical placements.

Interestingly, there were some positive results such as a decline in smoking, with a suggestion that some daily smokers may have been reducing smoking frequency. While this was not evident among the sub-group of 51 students, in a separate (as yet unpublished) study some referred to the striking impact of observing the direct effects of smoking in clinical practice which may be a cue for action to behaviour change. This is important as meta-analysis indicates that smoking in nursing students is high worldwide [17]. However, in a study in Spain and Portugal the proportions of students smoking increased with each successive year [18]. Further research is needed on how to reverse this trend.

This study relied upon the use of a self-report questionnaire, the limitation being that the accuracy of student responses cannot be guaranteed. Student responses may have been influenced by social desirability bias, and/or by an increased awareness of the importance of health behaviours as a result of their undergraduate training. Many of the questions categorised respondents into relatively wide categories, although the survey was intended to identify broad areas of concern warranting further investigation or intervention, for which very sensitive measures were not necessarily required. One weakness of the study was the lower response rate among third year students. The survey was administered during a timetabled session. Either student turn-out was particularly low for this session, or many students decided not to return their questionnaire. The question is whether non-response was associated with poorer self-rated health or health behaviours; in which case we might expect students in better health to be more likely to attend class and therefore observe improved health among the third year group; this did not appear to be the case. Although we identified 51 students for whom data were available at three time-points, patterns observed in cross-sectional analyses were not observed in this smaller sub-group for smoking and physical activity. It is important to note though that their health behaviours in Year 1 appeared more favourable than in the entire cohort, so they may not be representative of the entire cohort for health behaviours. In contrast, in their first year, their mean WEMWBS score (indicative of mental wellbeing), was similar to that of the overall cohort.

Our study has identified an important overall trend of declining mental health among undergraduate nursing students over time. Given the substantial body of evidence indicating that physical activity improves mental wellbeing [19, 20], it is likely that they would benefit from increased levels of physical activity.

## Conclusions

We recommend the incorporation of an intervention into the undergraduate nursing curriculum that promotes and encourages regular physical activity. This could offer students the opportunity to learn about health promotion and lifestyle change in practice to improve their own physical health, and to address mental wellbeing. With the increasing role nurses play in health promotion and calls for them to act as healthy role models for patients, it is vital that nursing students are supported to improve their own health from the beginning of their training.

## Supplementary Information

**Additional file 1.** Copy of administered questionnaire.

## Data Availability

The datasets used and/or analysed during the current study are available from the corresponding author on reasonable request.

## Abbreviations

HEI: Higher Education Institution
WEMWBS: Warwick-Edinburgh Mental Wellbeing Scale
BMI: Body Mass Index
