# Supplementary material for: How does the self-reported health of undergraduate nursing students change during their degree programme? Survey results from a Scottish University
Source: BMC Nurs. 2021 Mar 17;20:44. doi: 10.1186/s12912-021-00563-w (PMC7968279; doi:10.1186/s12912-021-00563-w)
Supplement: Supplementary file 1 — Additional file 1. Copy of administered questionnaire. [file 12912_2021_563_MOESM1_ESM.doc]

|  |  |  |
| --- | --- | --- |

**SECTION 1: Physical Activity**

If you would prefer to skip this section please tick here:

1. How would you rate your general physical health?

Excellent

Very Good

Good

Fair

Poor

2. How many hours do you spend sitting on a usual day?

Weekday

Weekend

3. In a normal week, on how many days are you physically active for a total of 30 minutes or more?

Note: Physical activity may include walking or cycling for recreation or to get to and from places; gardening; and exercise or sport.

0-2 days a week

3-4 days a week

5 or more days a week

**If you answered c. to question 3 skip to question 5.**

4. Have you been physically active for at least two and half hours in total over the course of the past week?

Yes

No

5. Have you become more physically active over the last year?

More active

Less active

No change

6. Would you like to be more physically active?

Yes

No

`

7. If yes, have you made any plans about how you might become more active, and what are these plans?

B

**SECTION 2: Eating Habits**

If you would prefer to skip this section please tick here:

8. How healthy do you consider your eating habits to be?

Very Healthy

Healthy

Average

Unhealthy

Very Unhealthy

9. Do you have breakfast most days?

Yes

No

10. How many portions of fruit and vegetables do you eat in a typical day?

Note: One portion is approximately one piece of medium-sized fruit or 80g of any one vegetable

0

1

2

3

4

5

6 or more

11. How many caffeinated, fizzy, ‘energy drinks’ do you consume during an average week? By ‘energy drinks’ we mean drinks that are marketed to give you a boost eg Red Bull, Loaded *etc*

I never drink them

Fewer than one per week

1-3 per week

4-7 per week

8-10 per week

More than 10

12. How often do you have a take-away, or eat in a fast food restaurant (eg McDonalds), for one of your main meals (breakfast, lunch or dinner)?

a. Every day

b. Most days

c. Once or twice a week

d. Occasionally (less than once a week)

**SECTION 3: Sleep**

If you would prefer to skip this section tick here:

13. In general, how well do you sleep?

Very well

Well

Average

Badly

Very Badly

14. How many hours of sleep do you get on an average night?

Less than 4

4-6

7-8

9-10

More than 10

15. At what time of day do you usually have your last caffeinated drink?

Note: Drinks with caffeine include tea, coffee, fizzy drinks such as cola and energy drinks.

16. How long do you spend looking at a smartphone, tablet or laptop in bed before you go to sleep (apart from setting your alarm)?

I don’t look at my smartphone/tablet/laptop in bed before I go to sleep

Up to 5 minutes

5 minutes to half an hour

Half an hour to an hour

An hour or more

**SECTION 4: Alcohol**

If you would prefer to skip this section tick here:

17. Do you consume alcoholic drinks?

Yes – every day

Yes – most days

Yes – once or twice a week

Yes – only occasionally

No - never

**If no, go to question 26 in Section 5.**

**1**8. What is the average number of alcohol units you consume per week?

Note: A pint of 5.2% lager/beer/cider has 3 units of alcohol; A standard 175ml glass of wine has 2.1 units, a 330ml bottle of lager/beer/cider has 1.7 units; a 275ml bottle of alcopops such as Blue WKD has 1.5 units; a single 25ml measure of spirits has 1 unit.

19. How many units of alcohol do you drink on a typical day when you are drinking?

1-2

3-4

5-6

7-9

10+

20. How often do you have 6 (if female)/8 (if male) or more alcohol units on a single occasion?

Never

Less than monthly

Monthly

Weekly

Daily or almost daily

21. How often do you drink alcohol at home (pre’s/pre-loading) before going on a night out?

Never

Less than monthly

Monthly

Weekly

Daily or almost daily

22. How many units of alcohol do you drink on a typical occasion when you have pre’s/are pre-loading?

None – I don’t have pre’s/preload

1-2

3-6

7-9

10+

23. How often in the last year, have you had a feeling of regret or guilt after drinking?

Daily or almost daily

Weekly

Monthly

Less than monthly

Never

24. How often in the last year, have you been unable to remember what happened the night before because you had been drinking?

Daily or almost daily

Weekly

Monthly

Less than monthly

Never

**SECTION SIX: Smoking and Vaping**

**SECTION 6: Smoking and Vaping**

25. Would you like to drink less alcohol?

Yes

No

Maybe

**SECTION 5: Smoking**

If you would prefer to skip this section tick here:

26. Which of the following statements best describes you?

I have never smoked

I used to smoke but I have now given up

I smoke but not everyday

I smoke everyday

**If you answered a or b go to question 32 in Section 6**

27. Which of the following do you smoke? (Circle all that apply)

Cigarettes

Roll-ups

e-cigarettes / vaping

Other. Please state:

28. How often do you smoke?

**Cigarettes/Roll upsE-cigarettesOther**Once a month

Once a month

Once a month

Once a fortnight

Once a fortnight

Once a fortnight

Once a week

Once a week

Once a week

Twice a week

Twice a week

Twice a week

Three times a week

Three times a week

Three times a week

Everyday or nearly everyday

Never

Everyday or nearly everyday

Never

Everyday or nearly everyday

Never

**SECTION SEVEN: Mental Health**

29. How many cigarettes/roll-ups have you smoked over the past week? (Do not include vaping)

0

1-10

11-20

21-50

More than 50

30. Have you started smoking, or started smoking more, in the last year?

Yes

No

31. Would you like to give up smoking?

Yes

No

Maybe

**SECTION 7: Mental Health**

**SECTION 6: Mental Health**

If you would prefer to skip this section please tick here:

32. How would you rate your general mental health?

Note: According to the World Health Organization, mental health is defined as a state of well-being in which every individual realizes his or her own potential, can cope with the normal stresses of life, can work productively and fruitfully, and is able to make a contribution to her or his community.

Excellent

Very good

Good

Fair

Poor

33. In the last year, have you ever had an emotional/mental health difficulty that has affected your life/studies?

Yes

No

Thank you for taking the time to complete this questionnaire!

If this questionnaire makes you worried in any way, you can contact the University of Stirling Counselling and Wellbeing Service: student.counselling @stir.ac.uk

If you want more information on a healthy lifestyle, you can look at the NHS Live Well site:

<http://www.nhs.uk/LiveWell/Pages/Livewellhub.aspx>

34. Please tick the box that best describes your experience of each over the last 2 weeks:

**None of the TimeRarelySome of the TimeOftenAll of the Time**

I’ve been feeling optimistic about the future

12345I’ve been feeling useful

12345I’ve been feeling relaxed

12345I’ve been feeling interested in other people

12345I’ve had energy to spare

12345I’ve been dealing with problems well

12345I’ve been thinking clearly

12345I’ve been feeling good about myself

12345I’ve been feeling close to other people

12345I’ve been feeling confident

12345I’ve been able to make up my own mind about things

12345I’ve been feeling loved

12345I’ve been interested in new things

12345I’ve been feeling cheerful

12345

**SECTION 7: About You**

35. What age are you?

36. What is your gender Male Female Non-binary

37. Would you describe yourself as a disabled person? Yes No Prefer not to say

Note: The Equality Act 2010 defines a disabled person as anyone who has or has had a physical or mental impairment which has a substantial and long-term effect on their ability to carry out normal day-to-day activities.

38. What is your height? What is your weight?

39. Are you (or any of your siblings) the first person to go to University in your family?

YesNo
